# Impact of the presence of sinus rhythm during cavotricuspid isthmus ablation for atrial flutter on the incidence of future new-onset atrial fibrillation: insights from an international multi-centre registry

**DOI:** 10.1093/europace/euaf039

**Published:** 2025-02-28

**Authors:** Josip Katic, Patrick Badertscher, Ivan Zeljkovic, Peter Ammann, Tobias Reichlin, Sven Knecht, Philipp Krisai, Michael Kühne, Christian Sticherling

**Affiliations:** Department of Cardiology, University Hospital Basel, University of Basel, Petersgraben 4, CH-4059 Basel, Switzerland; Cardiovascular Research Institute Basel, University Hospital Basel, Spitalstrasse 2, CH-4056 Basel, Switzerland; Department of Cardiology, University Hospital Split, Split, Croatia; Department of Cardiology, University Hospital Basel, University of Basel, Petersgraben 4, CH-4059 Basel, Switzerland; Cardiovascular Research Institute Basel, University Hospital Basel, Spitalstrasse 2, CH-4056 Basel, Switzerland; Department of Cardiology, University Hospital Sestre Milosrdnice, Zagreb, Croatia; Department of Cardiology, Cantonal Hospital St. Gallen, St. Gallen, Switzerland; Department of Cardiology, Inselspital, Bern University Hospital, Bern, Switzerland; Department of Cardiology, University Hospital Basel, University of Basel, Petersgraben 4, CH-4059 Basel, Switzerland; Cardiovascular Research Institute Basel, University Hospital Basel, Spitalstrasse 2, CH-4056 Basel, Switzerland; Department of Cardiology, University Hospital Basel, University of Basel, Petersgraben 4, CH-4059 Basel, Switzerland; Cardiovascular Research Institute Basel, University Hospital Basel, Spitalstrasse 2, CH-4056 Basel, Switzerland; Department of Cardiology, University Hospital Basel, University of Basel, Petersgraben 4, CH-4059 Basel, Switzerland; Cardiovascular Research Institute Basel, University Hospital Basel, Spitalstrasse 2, CH-4056 Basel, Switzerland; Department of Cardiology, University Hospital Basel, University of Basel, Petersgraben 4, CH-4059 Basel, Switzerland; Cardiovascular Research Institute Basel, University Hospital Basel, Spitalstrasse 2, CH-4056 Basel, Switzerland

**Keywords:** Typical atrial flutter, Atrial fibrillation, Radiofrequency, Ablation, Cavotricuspid isthmus, Oral anticoagulation

## Introduction

Typical atrial flutter (AFL) is a macroreentrant tachycardia involving the cavotricuspid isthmus (CTI) as a critical component of its circuit. Radiofrequency catheter ablation of the CTI (RFA-CTI) is a highly effective and safe treatment for AFL.^1^ However, individuals who have undergone successful AFL ablation are at higher risk for future atrial fibrillation (AF), with reported AF incidence rates ranging from 18% to 50%.^2^ Predicting future AF is crucial for determining the need for on-going oral anticoagulation (OAC) following CTI ablation. This study investigates whether the presence of AFL during RFA-CTI is associated with the occurrence of new-onset AF.

## Methods

We conducted a retrospective analysis using data from the multi-centre BEAT-Flutter registry, which included patients undergoing CTI-dependent AFL ablation at five centres in Switzerland and Croatia between January 2017 and January 2023. Patients with a history of AF, non-CTI-dependent AFL, or concurrent pulmonary vein isolation (PVI) were excluded. In patients presenting in sinus rhythm (SR), a cardiologist reviewed all recorded electrocardiograms (ECGs) and only those with documented typical AFL were included. In patients presenting in AFL entrainment from the tricuspid annulus was performed and CTI-dependent AFL was diagnosed if atrial activation was identical during entrainment and the post-pacing interval was ≤ 30 milliseconds (ms) or if the arrhythmia terminated during ablation. Patients underwent RFA-CTI under deep sedation using a 3.5 mm irrigated-tip catheter with 30–40 Watts to create a linear lesion from the tricuspid annulus to the inferior vena cava. Bidirectional conduction block confirmed procedural success. Clinical assessment with 12-lead ECG and 24-h Holter monitoring was performed at 3, 6, and 12 months post-ablation, with additional ECGs obtained for symptomatic episodes. The primary outcome was incident AF, detected in a 12-lead ECG or Holter-ECG after RFA-CTI. Secondary endpoints included recurrence of AFL, symptoms associated with any occurring arrhythmia (AF of AFL), and stroke or transient ischaemic attack (TIA) during follow-up.

## Results

**Figure 1:**
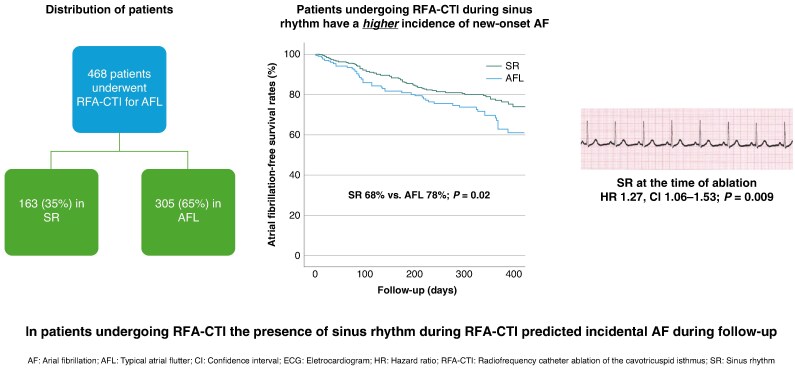
Impact of the presence of sinus rhythm during cavotricuspid isthmus ablation on the incidence of future new-onset atrial fibrillation.

Among 949 patients undergoing RFA-CTI, 50% were excluded due to a history of AF or missed follow-up. Of the remaining 468 patients, 65% underwent ablation during AFL and 35% during SR. Mean age was 68 ± 10 years, 5% were female, and the median CHA2DS2-VASc score was 2 (IQR 1–3). Bidirectional conduction block was achieved in all cases without significant differences in baseline or procedural parameters, as well as complications between groups. The median follow-up was 359 days (IQR 245–396). In total, 87% of patients had a follow-up 3 months after the procedure, 66% 6 months and 74% 1-year post-ablation. A total of 206 patients completed all defined follow-ups. Holter monitoring was available on 81% of patients at three months post- and 70% at both 6- and 12-months post-index procedure. New-onset AF occurred in 25% of patients (*n* = 119), with symptoms reported in only 59% of these cases. The median time to first AF episode was 141 days (IQR 81–277). AF-free survival was significantly higher in the AFL group compared to the SR group (78% vs. 69%; *P* = 0.02) (*Figure 1*). Median time to first AF episode was 154 days (IQR 83–244) in the AFL group and 124 days (IQR 77–280) in the SR group. In multi-variate analysis the presence of SR during RFA-CTI predicts future new-onset AF (HR 1.27, CI 1.06-1.53; *P* = 0.009), but there was no difference in recurrence rates of AFL (4% vs. 6%; *P* = 0.36). During follow-up, seven patients (1.5%) experienced a stroke or TIA, with most events occurring on OAC.

## Discussion

Our findings confirm a 25% incidence of new-onset AF following RFA-CTI for AFL in patients without a history of AF. This is in line with previous studies with incidence rates of up to 82% after 3 years in different populations.^3^ Importantly, we show for the first time, that the presence of on-going AFL during CTI ablation is associated with a lower incidence of future new-onset AF when compared to patients undergoing ablation during SR. One possible explanation is that some patients undergoing RFA-CTI in SR may not have had CTI-dependent AFL but pseudoregularized AF, atrial, left atrial flutter or incorrect ECG interpretations, which were misdiagnosed as atrial flutter.^4^ Waldo et al. suggested that in many cases short episodes of rapid AF precede the onset of AFL. The pivotal electrophysiological determinant influencing the transition into sustained AFL lies in the establishment of functional refractoriness or unidirectional block between the superior and inferior vena cava along the crista terminalis.^5^ The recently published CRAFT trial corroborated this hypothesis by demonstrating that cryoballoon PVI without CTI as first-line treatment for AFL is equally effective as standard CTI.^6^ During follow-up, a total of 1.5% of patients experienced a stroke, which was in the realm of patients in the seminal randomized controlled DOAC trials in AF patients. Stroke ranged between 1.11% and 1.7% per year on a DOAC.^7–10^ Of note, except two patients all patients with a stroke were on continued OAC. Notably, 41% of patients with new-onset AF were asymptomatic, underscoring the importance of comprehensive follow-up after successful ablation of AFL.

This study has limitations. First, the observational nature of our study precludes establishing causality. Secondly, the 1-year follow-up may have missed late occurrences of AF, and the non-continuous monitoring might have overlooked asymptomatic episodes. Another limitation is the incomplete follow-up in some patients, due to the COVID-19 pandemic.

## Conclusions

Every fourth patient without a history of AF undergoing RFA-CTI for AFL will develop new-onset AF during a 1 year follow-up and 41% of these are asymptomatic. The presence of SR during RFA-CTI increases the risk for the occurrence of new-onset AF during the one year follow-up. This has important implications when deciding about the termination of OAC after successful ablation of AFL.

## Data Availability

Data are available from the authors upon request.
